# The criteria for metabolic syndrome and the national health screening and education system in Japan

**DOI:** 10.4178/epih.e2017003

**Published:** 2017-01-06

**Authors:** Kazumasa Yamagishi, Hiroyasu Iso

**Affiliations:** 1 Department of Public Health Medicine, Faculty of Medicine, University of Tsukuba, Tsukuba, Japan; 2 Public Health, Department of Social Medicine, Osaka University Graduate School of Medicine, Suita, Japan

**Keywords:** Metabolic syndrome X, Universal health screening, Health education, Risk factors, Abdominal obesity

## Abstract

Two major definitions of metabolic syndrome have been proposed. One focuses on the accumulation of risk factors, a measure used by the American Heart Association (AHA) and the National Heart, Lung, and Blood Institute (NHLBI); the other focuses on abdominal obesity, a measure used by the International Diabetes Federation (IDF) and the Japanese government. The latter definition takes waist circumference (WC) into consideration as an obligatory component, whereas the former does not. In 2009, the IDF, NHLBI, AHA, and other organizations attempted to unify these criteria; as a result, WC is no longer an obligatory component of those systems, while it remains obligatory in the Japanese criteria. In 2008, a new Japanese cardiovascular screening and education system focused on metabolic syndrome was launched. People undergoing screening are classified into three groups according to the presence of abdominal obesity and the number of metabolic risk factors, and receive health educational support from insurers. This system has yielded several beneficial outcomes: the visibility of metabolic syndrome at the population level has drastically improved; preventive measures have been directed toward metabolic syndrome, which is expected to become more prevalent in future generations; and a post-screening education system has been established. However, several problems with the current system have been identified and are under debate. In this review, we discuss topics related to metabolic syndrome, including (1) the Japanese criteria for metabolic syndrome; (2) metabolic syndrome and the universal health screening and education system; and (3) recent debates about Japanese criteria for metabolic syndrome.

## INTRODUCTION

Japan has several unique points in criteria, screening and education systems for metabolic syndrome. In this review, we discuss topics related to metabolic syndrome in Japan, including the Japanese criteria for metabolic syndrome; the universal health screening and education system for metabolic syndrome; and recent debates about Japanese criteria for metabolic syndrome.

## JAPANESE CRITERIA FOR METABOLIC SYNDROME

**Figure 1.**
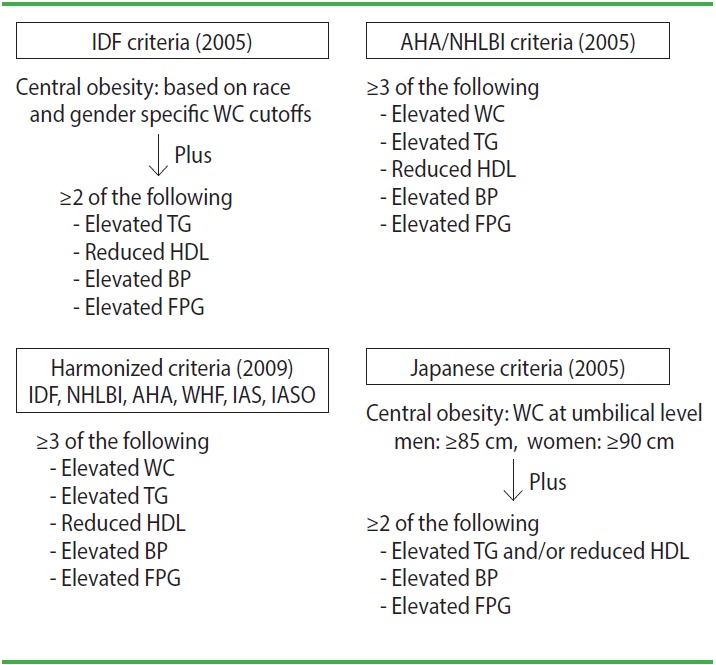
Selected criteria of metabolic syndrome. IDF, International Diabetes Federation; WC, waist circumference; TG, triglyceride; HDL, high-density lipoprotein cholesterol; BP, blood pressure; FPG, fasting plasma glucose; AHA, American Heart Association; NHLBI, National Heart Lung and Blood Institute; WHF, World Heart Federation; IAS, International Atherosclerosis Society; IASO, International Association for the Study of Obesity; BMI, body mass index; SBP, systolic blood pressure; DBP, diastolic blood pressure.

Many definitions of metabolic syndrome have been used worldwide, but they can be classified into two major types of criteria. The definition of the International Diabetes Federation (IDF) represents one type of criteria [1], while the definitions of the American Heart Association (AHA) and the National Heart, Lung and Blood Institute (NHLBI) of the US represent the other type (Figure 1) [2]. The major difference between these definitions is that the IDF criteria regard central obesity as an essential component of metabolic syndrome, whereas the AHA criteria focus on the accumulation of metabolic risk factors. The AHA criteria take waist circumference (WC) into consideration as one of five risk factors and define metabolic syndrome as the presence of any three components out of these five. The Japanese criteria [3] were established in 2005 and are quite similar to those of the IDF, except for the cutpoint for WC (Figure 1).

In 2009, the IDF, NHLBI, AHA, and other organizations unified their criteria [4], and these newer criteria are essentially similar to the AHA/NHLBI criteria (Figure 1). As a result, only the Japanese criteria maintain WC as an essential component of the definition.

The prevalence of metabolic syndrome among the Japanese according to different criteria was compared in a community sample from the Circulatory Risk in Communities Study (1990-1993, Kyowa, Japan; subjects aged 40-69 years old) [5]; 33% were evaluated by the AHA/NHLBI criteria; 27% were evaluated by the IDF criteria; and 17% were evaluated by the Japanese criteria.

## METABOLIC SYNDROME AND THE UNIVERSAL HEALTH SCREENING AND EDUCATION SYSTEM

Before the 1950s, the leading cause of death in Japan was tuberculosis, and the main measure at that time was the universal tuberculosis screening system. From the 1950s to 1980s, the leading cause was stroke; thus, the national prevention strategy focused on high blood pressure screening. In 1981, cancer overtook stroke, and in 1985 heart disease also overtook stroke, followed by pneumonia in 2011. Stroke is now the fourth leading cause of death in Japan. Given these changes, the Japanese government has shifted the target of screening to metabolic syndrome for the prevention 2and control of diabetes mellitus and the prevention of coronary heart disease and chronic kidney disease. In 2008, the Japanese government launched a new cardiovascular screening and education system, focusing on the detection and control of metabolic syndrome.

This policy change was informed by the existence of two major types of arteriosclerosis: atherosclerosis, which affects large arteries such as the coronary artery and middle cerebral arteries; and arteriolosclerosis, which affects small vessels such as the perforating arteries in the brain [6]. Atherosclerosis predominates in Americans and Europeans, reflecting the greater prevalence of dyslipidemia, glucose abnormalities, and metabolic syndrome, whereas arteriolosclerosis predominates in East Asians, reflecting the greater prevalence of hypertension [6].

The prevalence of hypertension and arteriolosclerosis has decreased over the past 50 years in Japan, and lifestyle patterns in East Asia are undergoing rapid westernization, especially in the middle-aged urban male population. This is the rationale that the Japanese government considered for changing the target of preventive screening to metabolic syndrome. In addition, although Japan has already established a universal health screening system, a post-screening education system has not been sufficiently developed. WC can function as a visible marker for motivating people to make lifestyle changes after screening, which is another rationale for the government to target metabolic syndrome.

**Figure 2.**
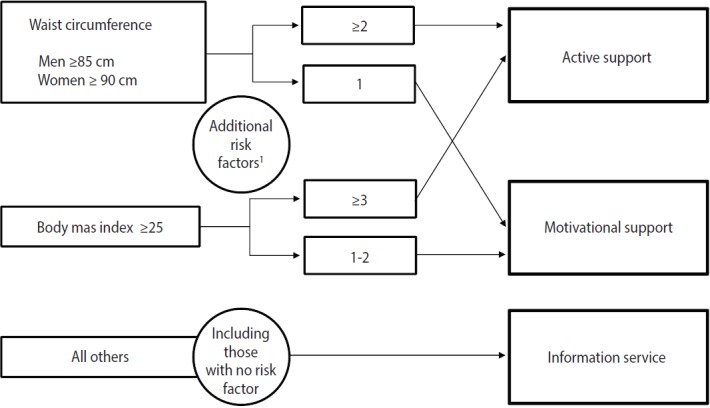
Current system of post-screening measures for metabolic syndrome. ^1^Additional risk factors: hyperglycemia (FBS ≥100 mg/dL or HbA1c ≥5.6%); dyslipidemia (TG ≥150 mg/dL or HDL ≤40 mg/dL); hypertension (SBP ≥130 mmHg or DBP ≥ 85 mmHg); Smoking. FBS, fasting blood sugar; HbAlc, glycated hemoglobin; TG, triglyceride; HDL, high-density lipoprotein cholesterol; SBP, systolic blood pressure; DBP, diastolic blood pressure.

The current system of post-screening measures for metabolic syndrome is shown in Figure 2. Based on the results of the screening, participants are classified into three groups based on the criteria for metabolic syndrome. As previously stated, WC is an essential component of the criteria for metabolic syndrome, and if participants exhibit two or more other risk factors, the insurer provides them with active support for making changes to their lifestyle. If they have abdominal obesity and one risk factor, then they are assigned to a motivational support group. All other participants receive information on preventing metabolic syndrome.

On the one hand, this system has produced several beneficial outcomes: the visibility of metabolic syndrome at the population level has drastically improved (although many people still confuse metabolic syndrome with simple obesity); preventive measures have been directed toward metabolic syndrome, which is expected to become more prevalent in future generations; and a post-screening education system has been established. On the other hand, several problems with this system have been identified, which are now under debate.

## RECENT DEBATES ON PROBLEMS WITH THE JAPANESE UNIVERSAL HEALTH SCREENING AND EDUCATION SYSTEM FOR METABOLIC SYNDROME

Debate has arisen concerning the preventive system targeting metabolic syndrome and revisions to the program are currently under consideration. The following questions have informed the discussions of the Japanese government commission on the way of universal cardiovascular screening and education system.

### Q1: Is metabolic syndrome really important among the Japanese?

This question has arisen because many studies have shown that the population attributable risk fraction (PAF) of metabolic syndrome is not as high as the PAF of high blood pressure. For example, the Japan Public Health Center-based Prospective Study reported that the PAF of high blood pressure was 50% for men and 53% for women, whereas that of metabolic syndrome (based on IDF criteria) was 10% for men and 6% for women [7].

However, an upward trend in obesity among East Asian men (but not among women, and not as steep as among non-Asians) may increase the impact of metabolic syndrome in the near future, especially among younger men. Therefore, it has been decided that metabolic syndrome is of importance among the Japanese, as a matter of disease management and prevention of cardiovascular disease in the future.

### Q2: Is it appropriate to keep waist circumference as an essential component of the criteria for metabolic syndrome?

This question is based on the fact that many studies have shown that the PAF of metabolic syndrome itself is not as high as that found in the non-obese, but high-risk population. For example, the Ibaraki Prefectural Health Study reported that the PAF of cardiovascular mortality was 10% for persons with a body mass index (BMI) of ≥25 kg/m^2^ and two or more metabolic risk factors, whereas it was 15% for those with a BMI of <25 kg/m^2^ and two or more metabolic risk factors [8].

In response, the committee has determined that it is necessary to provide additional health education for non-obese, but high-risk people.

### Q3: Are the Japanese waist circumference cutpoints appropriate?

The harmonized criteria of metabolic syndrome define central obesity with race-specific and gender-specific WC cutoffs [4]. Japanese cutoffs are ≥85 cm for men and ≥90 cm for women, defined based on cross-sectional studies that showed that these cutpoints were equivalent to 100 cm^2^ of visceral fat area [3]. These cutoffs have sometimes been criticized, because only Japan sets higher cutoffs for women than men. However, according to a study supported by the Ministry of Health, Labour, and Welfare [9], changing WC cutoffs from 80 to 90 cm had virtually no effect on the impact of metabolic syndrome on cardiovascular disease. Taken together with the fact that the incidence of cardiovascular disease is much lower in women than in men, the committee concluded that the WC cutpoints should be maintained as is.

## CONCLUSION

The conclusions of this review are as follows. First, the Japanese criteria for metabolic syndrome differ in several respects from the international criteria, including the use of WC as an essential component, with unique WC cutpoints. Second, though a universal metabolic syndrome screening and education system was launched in 2008, the epidemiological evidence for the metabolic syndrome measures was not sufficient at the start of the system’s implementation. Third, revision of the metabolic syndrome screening system is now under consideration, but it is unlikely to be changed drastically. Fourth, considering measures of metabolic syndrome in East Asia, we should note that the etiology of arteriosclerosis differs between East Asians and Westerners.
